# The Transcriptional Signature of Active Tuberculosis Reflects Symptom Status in Extra-Pulmonary and Pulmonary Tuberculosis

**DOI:** 10.1371/journal.pone.0162220

**Published:** 2016-10-05

**Authors:** Simon Blankley, Christine M. Graham, Jacob Turner, Matthew P. R. Berry, Chloe I. Bloom, Zhaohui Xu, Virginia Pascual, Jacques Banchereau, Damien Chaussabel, Ronan Breen, George Santis, Derek M. Blankenship, Marc Lipman, Anne O’Garra

**Affiliations:** 1 Laboratory of Immunoregulation and Infection, The Francis Crick Institute, Mill Hill Laboratory, London, United Kingdom; 2 Baylor Research Institute, Baylor Scott & White, Dallas, Texas, United States of America; 3 Department of Respiratory Medicine, Imperial College Healthcare NHS Trust, St Mary’s Hospital, London, United Kingdom; 4 Baylor Institute for Immunology Research/ANRS Center for Human Vaccines, INSERM, Dallas, Texas, United States of America; 5 The Jackson Laboratory for Genomic Medicine, 10 Discovery Drive, Farmington, 06032, Connecticut, United States of America; 6 Sidra Medical and Research Center, Doha, Qatar; 7 Department of Respiratory Medicine, King’s College London, London, United Kingdom; 8 Division of Asthma, Allergy and Lung Biology, King’s College London, London, United Kingdom; 9 Department of Respiratory Medicine, Royal Free London NHS Foundation Trust, London, United Kingdom; 10 Division of Medicine, University College London, London, United Kingdom; 11 Department of Medicine, NHLI, Imperial College, London, United Kingdom; Institut de Pharmacologie et de Biologie Structurale, FRANCE

## Abstract

**Background:**

*Mycobacterium tuberculosis* infection is a leading cause of infectious death worldwide. Gene-expression microarray studies profiling the blood transcriptional response of tuberculosis (TB) patients have been undertaken in order to better understand the host immune response as well as to identify potential biomarkers of disease. To date most of these studies have focused on pulmonary TB patients with gene-expression profiles of extra-pulmonary TB patients yet to be compared to those of patients with pulmonary TB or sarcoidosis.

**Methods:**

A novel cohort of patients with extra-pulmonary TB and sarcoidosis was recruited and the transcriptional response of these patients compared to those with pulmonary TB using a variety of transcriptomic approaches including testing a previously defined 380 gene meta-signature of active TB.

**Results:**

The 380 meta-signature broadly differentiated active TB from healthy controls in this new dataset consisting of pulmonary and extra-pulmonary TB. The top 15 genes from this meta-signature had a lower sensitivity for differentiating extra-pulmonary TB from healthy controls as compared to pulmonary TB. We found the blood transcriptional responses in pulmonary and extra-pulmonary TB to be heterogeneous and to reflect the extent of symptoms of disease.

**Conclusions:**

The transcriptional signature in extra-pulmonary TB demonstrated heterogeneity of gene expression reflective of symptom status, while the signature of pulmonary TB was distinct, based on a higher proportion of symptomatic individuals. These findings are of importance for the rational design and implementation of mRNA based TB diagnostics.

## Introduction

*Mycobacterium tuberculosis* (*Mtb*) continues to be a significant cause of mortality and morbidity worldwide [1]. In 2013 there were 9 million cases of active tuberculosis (TB) resulting in 1.5 million deaths worldwide, of these active TB cases approximately 15% were notified as extra-pulmonary TB [1]. Common sites of extra-pulmonary TB infection include the pleura, lymph nodes, meninges, bone and genito-urinary tract [2]. Extra-pulmonary TB can be challenging to diagnose compared to pulmonary TB as it is often not considered during clinical evaluation and diagnostic samples can be more difficult to obtain [3, 4].

Whole genome expression analysis of human whole blood as well as peripheral blood mononuclear cells (PBMC) has been widely used to profile the host transcriptional response in active TB and identify potential biomarkers for use in diagnostics (reviewed in [5]). An interferon-dominated 393 transcript signature was identified from human whole blood which was present in active TB and absent in the majority of healthy and latently infected individuals and was shown to correlate with the extent of radiographic lung disease [6]. This finding of enrichment for interferon signalling has since been reported in multiple subsequent studies [7–11]. Other studies have, in addition to interferon signalling, identified immunological pathways that may be relevant to the pathogenesis of active TB such as TLR signalling, complement as well as enrichment for T- and B-cell function gene expression [6–9, 11–15]. Two large studies have included extra-pulmonary TB patients within their active TB cohort, but no sub-group analysis was undertaken to determine if there was a transcriptional signature unique to extra-pulmonary TB [16, 17]. Therefore the question of whether there is a difference in the blood transcriptional signature between pulmonary and extra-pulmonary TB has not been answered.

We recently published a 380 gene meta-signature (S1 Table) of active TB compared to healthy controls [18], which represents an attempt to identify the most consistently differentially expressed genes across the published publicly available datasets [18]. Similar to the individual published studies the 380 gene meta-signature was found to be enriched for immune response pathways including multiple pattern recognition receptors, cytokines, the inflammasome, complement and immunoglobulin pathways [18].

We herein used this published derived gene set as well as other transcriptional analysis tools to test similarities and differences between pulmonary TB and extra-pulmonary TB in a novel dataset and now report heterogeneity driven mainly by the extent of symptoms.

## Materials and Methods

### Ethics

This study was approved by the Central London 3 Research Ethics Committee (09/H0716/41). All participants gave written informed consent.

### Clinical cohort

Recruitment took place between 2011 and 2014. Sites for recruitment were the Royal Free and Guy’s & St Thomas’ Hospitals, London, United Kingdom. Healthy controls and the majority of pulmonary TB patients were chosen from an existing bank of globin reduced RNA samples. These subjects had consented to their samples being used in further approved studies. Patients identified from this bank were selected on cohort group, age, sex and ethnicity. Diagnosis of active TB was based on the following: positive mycobacterial culture result from the site of disease; or caseating granuloma on biopsy and/or clinical/radiological features consistent with active TB and a good response to therapy. Sarcoidosis patients all had mediastinal disease with compatible histology and clinical/radiological features to support a diagnosis of sarcoidosis and negative mycobacterial cultures. Patients were excluded if they were immunosuppressed either through disease (such as HIV, diabetes or autoimmune diseases), medication or had significant co-morbidities affecting the pulmonary system.

### Microarray

Human whole blood RNA was isolated, globin reduced and amplified as described previously [6, 19]. 750 ng of cRNA was hybridized to Illumina Human HT-12 V4 BeadChip arrays and scanned on Illumina iScan. GenomeStudio was used to perform quality control and generate signal intensity. Microarray analysis was undertaken using GeneSpring GX 13.0. Per sample normalisation (75^th^ centile) and per transcript normalisation (median of all samples) was performed. Microarray data were deposited in the NCBI Gene Expression Omnibus (GEO) with series accession number (GSE83456). All data collected and analysed in these experiments adhere to the Minimal Information About a Microarray Experiment (MIAME) guidelines.

### Expression analysis

Molecular Distance to Health (MDTH), was calculated using methodology previously described [20], for defined groups of genes (group of genes defined in legend of Figures) relative to a control group (detailed in legend of Figure). Additionally, the MDTH was calculated using the same methodology but now using 3409 transcripts, which represent the transcripts from 38 annotated modules [21]. Z-scores were derived from the respective MDTH’s and calculated relative to the control group.

Modular analysis was undertaken using log2 transformed normalised data [21].

Differentially expressed genes identified from new dataset for TB and sarcoidosis involved groups being compared to the healthy control cohort (transcripts filtered which were not significantly detected from background in at least 10% of samples, low expressed transcripts filtered (less than 2 fold change from median in 10% of samples), followed by statistical testing (independent t-test with Benjamini Hochberg multiple testing correction (*q-value* <0.05) between groups of interest). Transcripts were than matched to Entrez Gene identifiers; duplicates (retaining those with the largest fold change difference) and non-matched transcripts were filtered.

### Statistical analyses

GraphPad Prism 6 or Microsoft Excel (2010) were used for statistical analysis, details of statistical testing given in figure legends.

## Results

### Testing the meta-signature in a new dataset reveals differences in the transcriptional response of pulmonary and extra-pulmonary patients

**Fig 1:**
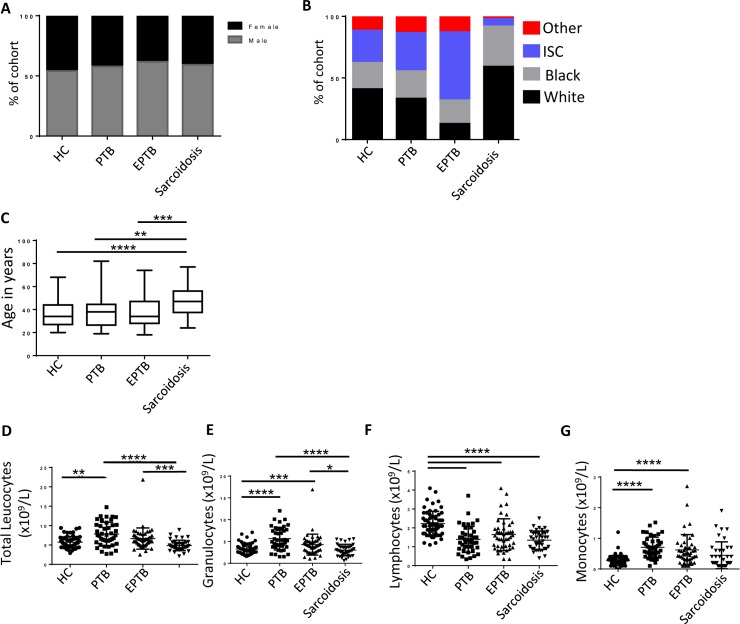
Clinical parameters of patients included in microarray dataset. **(A)** percentage of cohort by sex **(B)** percentage of cohort by ethnicity (ISC; Indian sub-continent) and **(C)** age (mean; min-max bars). Whole blood composition; **(D)** total leukocytes **(E)** Granulocytes **(F)** lymphocytes and **(G)** monocytes. Statistical tests: Kruskal Wallis with Dunn’s multiple testing correction.

A new cohort of extra-pulmonary and sarcoidosis patients was recruited together with an existing bank of pulmonary TB and healthy controls formed the dataset for analysis (Cohort details; S2 and S3 Tables). There was no difference in gender frequency (Fig 1A) between groups however there were differences in the group composition with regard to ethnicity and age, with the sarcoidosis group being significantly older than the other groups and tending to have less patients of Indian subcontinent background (Fig 1B and 1C). Total white cell count was significantly elevated in Pulmonary TB patients compared to the other groups (Fig 1D), this was mainly due to increased numbers of granulocytes compared to the other groups (Fig 1E). Total lymphocyte count was significantly higher in healthy controls compared to all the groups (Fig 1F) and monocytes were significantly elevated in Pulmonary and Extra-pulmonary TB patients compared to Healthy controls (Fig 1G).

**Fig 2:**
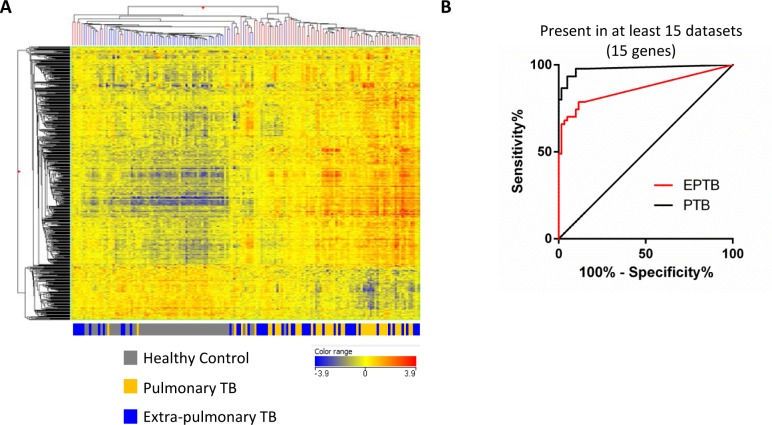
Testing the meta-signature and the most consistently identified genes in a new dataset. **(A)** The 380 gene meta-signature were mapped to 687 Illumina transcripts, of which 113 transcripts were excluded as they were not significantly detected from background in 10% of samples. Hierarchical clustering (Pearson uncentred (cosine) with averaged linkage) on individuals and transcripts broadly cluster healthy controls from pulmonary and extra-pulmonary TB patients. **(B)** Receiver operator curves for extra-pulmonary and pulmonary cohorts against healthy controls using MDTH derived from the most consistently identified genes (15 genes identified in at least 15 of the meta-analysis datasets [18]–representing the most robustly identified genes in that analysis, healthy controls used as control group for MDTH) as potential diagnostic biomarker. Both prediction results were validated using k-fold cross validation with k equal to 10 with 1,000 iterations. The mean AUC for the EPTB and PTB validation results are 0.865 (95% confidence interval: 0.857–0.872) and 0.977 (95% confidence interval: 0.974–0.981) respectively.

The previously defined 380 meta-signature genes [18] (S1 Table, mapped to 687 Illumina transcripts, of which 113 transcripts were excluded as they were not significantly detected from background in 10% of samples) were used for analysis in this new dataset. Hierarchical clustering of the dataset (healthy controls, pulmonary and extra-pulmonary TB patients only) revealed that the 380 meta-signature genes (S1 Table) were able to separate healthy controls from the majority of both pulmonary and extra-pulmonary TB patients, although a small number of TB patients clustered together with the healthy controls (Fig 2A). Using the most consistently identified genes from the meta-analysis as potential biomarkers (15 genes which were identified in 15 or more meta-analysis data-sets, S1 Table) and calculating a molecular score for these genes, there was a greater sensitivity in identifying pulmonary TB patients than extra-pulmonary TB patients from healthy controls (Fig 2B).

### Transcriptional signatures in TB reflect symptom status of individuals

**Fig 3:**
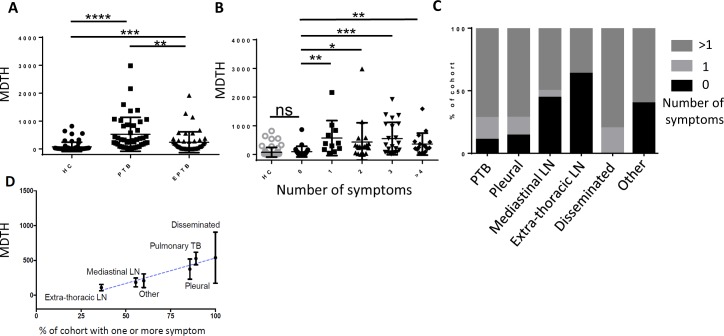
Molecular distance to health is linked to symptoms status and site of disease. (A) Molecular distance to health (MDTH) calculated for each individual (from 3409 transcripts which represent the transcripts of the 38 annotated modules shown in Fig 2A, healthy controls used as the control group). Individuals grouped by disease status, (median value, error bars SD) statistical testing Kruskal Wallis with Dunn’s multiple testing correction. **(B)** Patients grouped by number of reported symptoms; night sweats, fever, weight loss, chest pain or cough (median value, error bars SD) statistical testing Kruskal Wallis with Dunn’s multiple testing correction. **(C)** Patients grouped by site of disease and number of reported symptoms (as previous). **(D)** Mean MDTH (error bars, Standard error of the mean) plotted for each site of disease against % of the cohort suffering from one or more symptom (from list of symptoms described previously). Blue line represents Pearson’s correlation (R^2^ 0.95, *p* = 0.0090).

There was a significant difference in the molecular distance to health (MDTH) of both pulmonary TB and extra-pulmonary TB patients as compared to healthy controls (Fig 3A). Pulmonary TB had a significantly higher MDTH compared to extra-pulmonary TB (Fig 3A). The MTDH was not influenced by the ethnicity of the TB patients (S1A Fig) or by *M*. *tuberculosis* culture status in extra-pulmonary TB patients (S1B Fig).

Presence of one of the following symptoms: night sweats, fever, weight loss, chest pain or cough resulted in a significantly higher MDTH compared to healthy controls; there was no additive effect of increasing number of symptoms in terms of magnitude of MDTH (Fig 3B). Absence of any of the above symptoms resulted in no significant difference in MDTH score from healthy controls. There were differences in symptom prevalence depending on the site of disease (Fig 3C). The mean MDTH of the patients grouped by site of disease significantly correlated with the percentage of patients within the group having one or more symptom (Fig 3D). There was no difference in MDTH score of extra-pulmonary patients dependent on culture status (S1C Fig).

**Fig 4:**
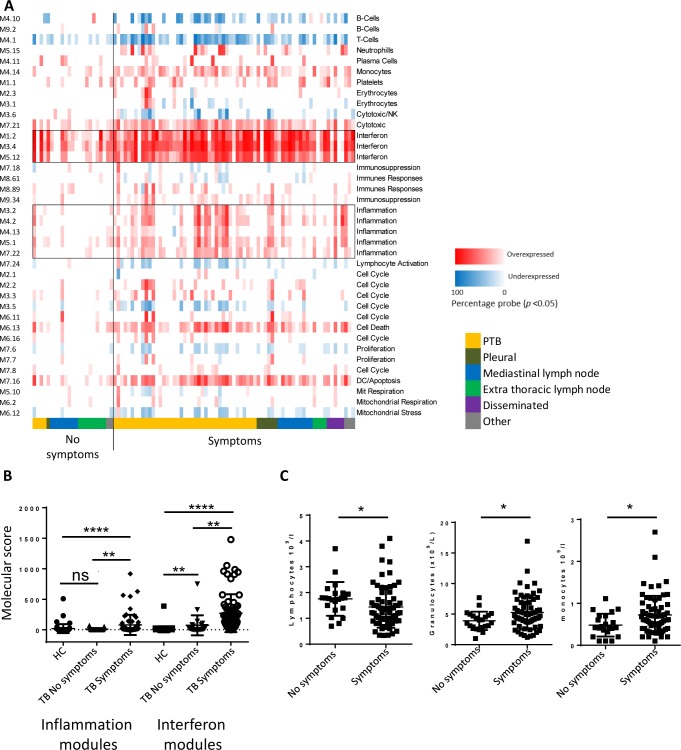
Symptom status linked to alteration in modular profile and blood counts. (A) Individual modular analysis for all TB patients grouped by symptom status and site of disease, boxed are modules annotated as interferon or inflammation. 38 annotated modules are displayed as a heatmap with red indicates significant over-abundance of transcripts and a blue indicating significant under-abundance (p <0.05). The colour intensity represents the percentage of genes in that module that are significantly differentially expressed. **(B)** MDTH calculated for all interferon module transcripts and all inflammatory module transcripts (healthy controls used as control group). TB patients grouped according to symptom status (median value, error bars SD) statistical testing Kruskal Wallis with Dunn’s multiple testing correction. **(C)** TB patients grouped according to symptom status with lymphocyte, granulocyte and monocytes counts shown (median value, error bars SD) statistical testing Mann-Whitney U.

Upon individual modular analysis, there was no apparent site-specific modular patter. Instead, presence of any of the five symptoms seemed to influence the modular pattern observed (Fig 4A). Molecular scoring of the genes within modules annotated as “inflammation” revealed no significant difference between healthy controls and the asymptomatic group of TB patients, in contrast to the significant difference between asymptomatic and symptomatic populations (Fig 4A and 4B). For the interferon modules there was a significantly higher score for the symptomatic group compared to the asymptomatic group (Fig 4A and 4B). While there were significantly higher lymphocyte and significantly lower granulocyte and monocyte counts in the asymptomatic group compared to the symptomatic TB patients, there was considerable overlap between the two populations (Fig 4C).

### Overlap in expression profiles between sarcoidosis and extra-pulmonary TB patients

**Fig 5:**
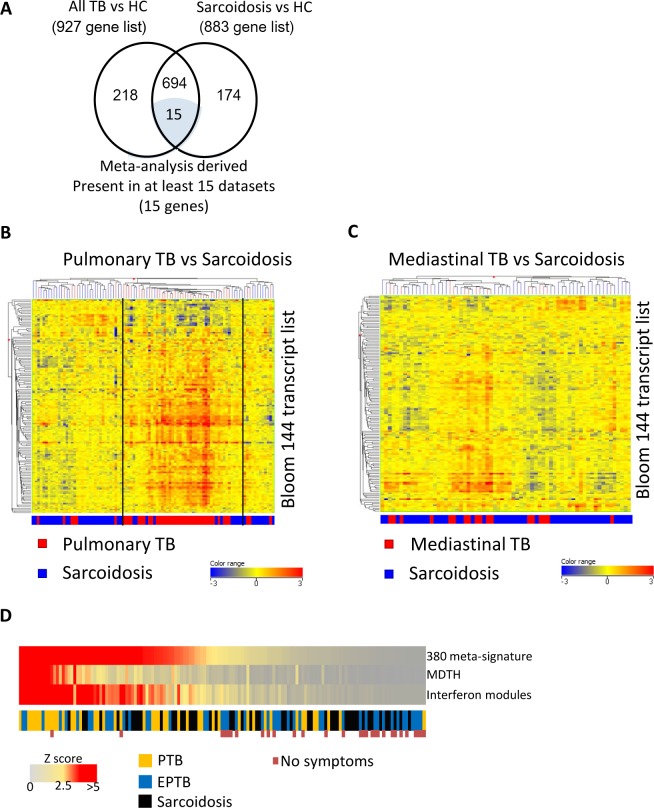
Tuberculosis and sarcoidosis have similar differentially regulated genes. **(A)** Differentially expressed genes identified from new dataset for TB and sarcoidosis groups compared to healthy controls (transcripts filtered which were not significantly detected from background in at least 10% of samples, low expressed transcripts filtered (less than 2 fold change from median in 10% of samples), followed by statistical testing (independent t-test with Benjamini Hochberg multiple testing correction (*q-value* <0.05) between groups of interest). Transcripts were matched to Entrez gene identifiers and duplicates and non-matched transcripts filtered. Venn diagram showing the overlap of DEGs between these two diseases and the most significant 15 gene list from the meta-analysis. **(B)** Heatmap of normalised expression using the Bloom et al 144 transcript list are able to broadly differentiate pulmonary TB patients from sarcoidosis patients. Clustering (Pearson’s uncentred (Cosine) with averaged linkage) on transcripts (rows) and individual patient blood samples (columns). **(C)** The same 144 transcript list (16) is unable to differentiate between mediastinal TB patients and sarcoidosis patients by clustering (as before) **(D)** Molecular scores calculated for transcripts from 380 gene meta-signature, MDTH and transcripts representing the interferon modules (healthy controls acting as control group). Z scores calculated (with healthy controls used as reference group for calculating mean and SD) and then TB patients and sarcoidosis patients ranked according to Z score of the 380 gene meta-signature. For all three outcomes the mean of pulmonary TB patients was significantly higher than both the extra-pulmonary TB and sarcoidosis patients (p<0.05). Differences among means were tested with a generalized linear model assuming a normal distribution and a Bonferroni multiple testing correction. No symptoms indicates absence of any of the five symptoms listed previously.

Analysis of all the TB patients from this new dataset identified 927 genes (S4 Table) as being differentially regulated compared to healthy controls and 883 genes (S4 Table) differentially regulated between sarcoidosis patients and healthy controls (Fig 5A). Overlapping these two gene lists revealed that 709 genes were commonly expressed between the two conditions compared to healthy controls, of which 15 had been previously identified from the meta-analysis as those most consistently expressed in TB (Fig 5A, S4 Table). Nine of the top 10 upregulated genes by fold change of TB compared with healthy controls were also found within the top 10 upregulated genes by fold change of sarcoidosis compared with healthy controls (Table 1).

**Table 1:** Top 10 differentially upregulated genes in TB and sarcoidosis^a^.

Tuberculosis		Sarcoidosis	
Gene	Log2 FC	Gene	Log2 FC
*SERPING1*	3.83	*ANKRD22*	3.35
*ANKRD22*	3.82	*SERPING1*	3.20
*BATF2*	3.61	*BATF2*	3.02
*FCGR1C*	3.04	*IFIT3*	2.67
*LOC728744*	3.04	*FCGR1A*	2.54
*GBP6*	3.03	*FCGR1C*	2.52
*C1QB*	3.02	*GBP6*	2.52
*FCGR1A*	3.02	*FCGR1B*	2.50
*FCGR1B*	2.90	*RAP1GAP*	2.44
*IFIT3*	2.84	*LOC728744*	2.42

^a^ Significantly upregulated genes as measured by fold change compared to healthy controls.

Of those genes which were identified in only TB or sarcoidosis compared with healthy controls, only one (*RNF182*) was two-fold different between TB and sarcoidosis (S4 Table). Using the *Bloom et al* 144 transcript list [19] by hierarchical clustering it was possible again to separate the majority of sarcoidosis patients from those with pulmonary TB (Fig 5B), but not from mediastinal TB (Fig 5C).

Calculating the molecular scores for the 380 meta-signature for all patients and ranking them in order of z score (compared to healthy controls) revealed that there was an overlap between the three groups, with pulmonary TB patients tending towards the higher end and extra-pulmonary and sarcoidosis patients the lower end of the spectrum (Fig 5D). Indeed, for all three outcomes the mean of pulmonary TB patients was significantly higher than both the extra-pulmonary TB and sarcoidosis patients. However, any individual regardless of disease type could lie anywhere within this range. This observation was consistent for MDTH and molecular scores for the interferon modules. Patients with none of the 5 listed symptoms, regardless of their disease, tended to have the lowest z scores (Fig 5D).

## Discussion

This study is the first to our knowledge to attempt to identify blood transcriptional signatures associated with the site of *Mtb* infection. We show that the transcriptional response is similar across sites of disease as measured from the blood although the magnitude of response varies and this is mainly associated with the presence or absence of symptoms and probably the site of the infection.

There were differences in the demographic composition of the cohorts in this study. Patients diagnosed with sarcoidosis were older and more likely to be of white or black ethnic background. This skewing in terms of age and ethnicity is well described in sarcoidosis [22, 23]. TB in the UK tends to be diagnosed from the immigrant population, in particular those from the Indian subcontinent [24], with extra-pulmonary TB being more common in those of Asian and African origin [25–27]. Hence the differences in ethnic composition between our clinical groups are likely to be due to a combination of these factors. These differences in group composition did not affect the transcriptional responses observed in our study. Similarly, whole blood cell composition in this study at the group level was found to be altered in pulmonary and extra-pulmonary TB compared to healthy controls with increased monocytes and granulocytes and decreased lymphocytes as has been previously described [28].

Extra-pulmonary TB represents *Mtb* infection which has spread haematogenously or via the lymphatic system from the lung. It is hypothesised that this spread happens during initial infection and that extra-pulmonary disease may represent reactivation rather than primary disease [4]. It has long been recognised that there is variation in symptoms and bacterial load dependent on site of disease, hence differences in the transcriptional signature may reflect variation in bacterial burden at the site of disease [4, 29–31], or in the site specific host immune response which may be reflected in the blood. Gene expression signatures of TB patients have previously been linked with bacterial burden, with a prior study showing reduced sensitivity of a whole genome expression derived disease risk score with decreased culture positivity in children [17]. Equally, with respect to complement which has been identified as one of the key findings in several microarray studies, *C1q* expression levels correlated with sputum smear positivity and diminished with treatment [12, 32]. Our findings of differences in MDTH (and symptoms) based on the site of disease may therefore be linked to differences in bacterial burden or the host site specific immune response. Integration of both host transcriptional response and more accurate quantitation of total mycobacterial burden may result in a better understanding of what drives the blood host transcriptional response.

Whilst it was possible to again here to distinguish pulmonary TB from sarcoidosis using our previously described gene set [19], it was not possible to distinguish sarcoidosis and mediastinal TB by hierarchical clustering. The inability to distinguish sarcoidosis and mediastinal TB, is possibly due to the lower symptom status of mediastinal extra-pulmonary TB cohorts versus pulmonary TB cohorts. This would fit with our findings that the magnitude of the transcriptional response as measured from the blood is mainly associated with the presence or absence of symptoms. Previous studies which had identified differentially expressed genes between sarcoidosis and TB had used only pulmonary TB patients as their comparator group [9, 19, 33]. We show that compared to healthy controls the gene expression signature of TB (including pulmonary and extra-pulmonary patients) is very similar to sarcoidosis. We show that individuals from each disease state (Pulmonary TB, Extra-pulmonary TB or sarcoidosis) can lie anywhere along this spectrum of gene expression including some clustering together with healthy controls. However when taken at the group level differences in magnitude of expression can be observed between pulmonary TB and the other disease states.

Both TB and sarcoidosis are granulomatous diseases, and together with another granulomatous disease melioidosis they have been shown to have similar gene expression signatures to TB [9, 19, 33, 34]. Despite these similarities, it may still be possible using clinical parameters and risk stratification to utilise mRNA expression based diagnostics to differentiate between diseases as has been done for active and latent TB [6–9, 15–18, 35].

We show here for the first time that blood based transcriptional signatures in pulmonary and extra-pulmonary TB differ as a result of the symptom status and site of the disease in each individual. These findings have implications for design and implementation of mRNA expression tools to support diagnostics and treatment monitoring of TB.

## Supporting Information

S1 Fig Molecular Distance to health. Molecular distance to health (MDTH) calculated for each individual (from 3409 transcripts which represent the transcripts of the 38 annotated modules shown in Fig 2A (Control group for molecular scores–healthy controls). **(A)** MDTH for TB patients by ethnicity (no significant difference between groups) (ISC; Indian sub-continent). **(B)** MDTH for extra-pulmonary patients–there was no significant difference in MDTH status (Mann-whitney U) dependent on culture status.
(TIFF)

S1 Table Identity of 380 genes expressed in blood of active TB patients from metaanalysis distinguish active TB patients from healthy Controls. List of genes identified in earlier study [18] now by by hierarchical clustering of the dataset (healthy controls, pulmonary and extra-pulmonary TB patients only) revealed that this 380 meta-signature genes separate healthy controls from the majority of both pulmonary and extra-pulmonary TB patients.
(XLSX)

S2 Table Numbers of patient within the cohorts of pulmonary TB, extra-pulmonary TB and sarcoidosis patients and healthy controls. (DOCX)

S3 Table Numbers of patients with different forms of TB in different body sites, their *Mtb* culture status, histology and clinical diagnosis status. (DOCX)

S4 Table Lists of 694 commonly expressed genes in the blood of TB and sarcoid patients as compared to healthy controls; unique TB genes and unique sarcoid genes expressed in blood as compared to healthy controls. (XLS)
