# Supplementary figures and images for: The Transcriptional Signature of Active Tuberculosis Reflects Symptom Status in Extra-Pulmonary and Pulmonary Tuberculosis
Source: PLoS One. 2016 Oct 5;11(10):e0162220. doi: 10.1371/journal.pone.0162220 (PMC5051928; doi:10.1371/journal.pone.0162220)

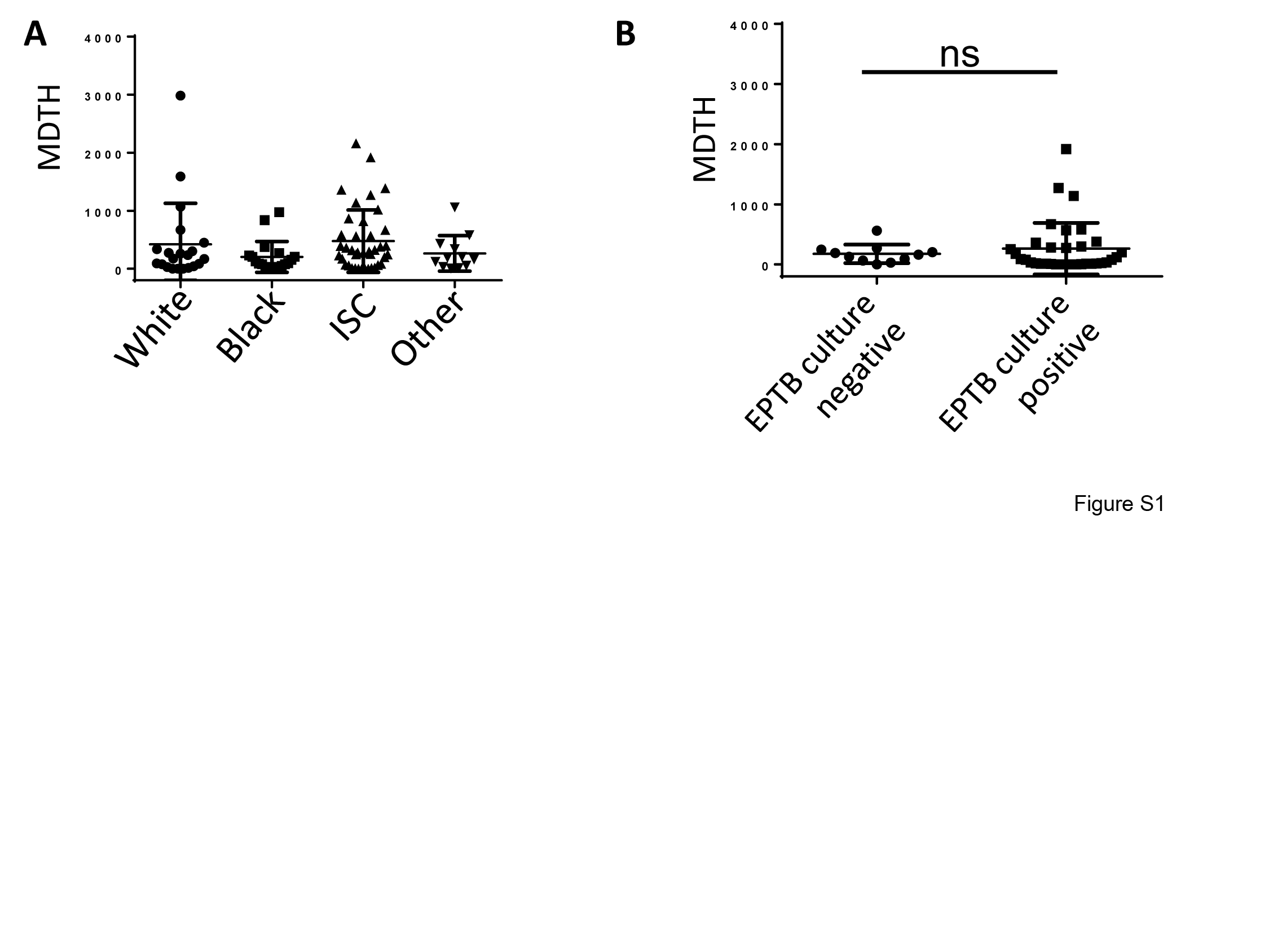

Supplement: S1 Fig — Molecular distance to health (MDTH) calculated for each individual (from 3409 transcripts which represent the transcripts of the 38 annotated modules shown in Fig 2A (Control group for molecular scores–healthy controls). (A) MDTH for TB patients by ethnicity (no significant difference between groups) (ISC; Indian sub-continent). (B) MDTH for extra-pulmonary patients–there was no significant difference in MDTH status (Mann-whitney U) dependent on culture status. (TIFF) [file pone.0162220.s001.tiff]
