# Supplementary material for: The Transcriptional Signature of Active Tuberculosis Reflects Symptom Status in Extra-Pulmonary and Pulmonary Tuberculosis
Source: PLoS One. 2016 Oct 5;11(10):e0162220. doi: 10.1371/journal.pone.0162220 (PMC5051928; doi:10.1371/journal.pone.0162220)
Supplement: S2 Table — (DOCX) [file pone.0162220.s003.docx]

**S2 Table:** Size of cohort in new microarray dataset

| Cohort | Number |
| --- | --- |
| Healthy controls | 61 |
| Pulmonary TB | 45 |
| Extra-pulmonary | 47 |
| Sarcoidosis | 49 |
|  | |
