# Supplementary material for: The Transcriptional Signature of Active Tuberculosis Reflects Symptom Status in Extra-Pulmonary and Pulmonary Tuberculosis
Source: PLoS One. 2016 Oct 5;11(10):e0162220. doi: 10.1371/journal.pone.0162220 (PMC5051928; doi:10.1371/journal.pone.0162220)
Supplement: S3 Table — (DOCX) [file pone.0162220.s004.docx]

| Site of TB | Total | Positive *Mtb culture* | Histological diagnosis | Clinical/  Radiological Diagnosis |
| --- | --- | --- | --- | --- |
| Pulmonary | 45 | 45 | 0 | 0 |
| Pleural | 7 | 7 | 0 | 0 |
| Mediastinal lymph node | 18 | 15 | 3 | 0 |
| Extra-thoracic lymph node | 12 | 7 | 5 | 0 |
| Disseminated (>2 sites) | 5 | 4 | 1 | 0 |
| Other (Abdominal (3), soft tissue (1) and bone (1)) | 5 | 3 | 0 | 2 |
|  | | | | |
